# Evaluation of CD56^dim^ and CD56^bright^ natural killer cells in peripheral blood of women with IVF failures

**Published:** 2015-09

**Authors:** Farahnaz Mardanian, Moones Kazeroonizadeh, Bahman Rashidi

**Affiliations:** 1 *Department of Obstetrics and Gynecology, School of Medicine, Isfahan University of Medical Sciences, Isfahan, Iran.*; 2 *Department of Anatomy, School of Medicine, Isfahan University of Medical Sciences,Isfahan, Iran.*

**Keywords:** *Natural killer cells*, *In vitro fertilization*, *Infertility*

## Abstract

**Background:**

Infertility is an increasing medical and social problem. In vitro fertilization (IVF) has become a common and accessible treatment for a wide variety of indications that have variable outcomes. Natural killer (NK) cells have been identified as relevant immunological factors involved in reproductive success or failure.

**Objective:**

The aim of this study was to compare the percentage of peripheral blood CD56^+^ (CD56^dim^ and CD56^bright^) cells and the level of NK cell in patients with IVF failure with those of successful IVF control women.

**Materials and Methods:**

We assessed the level of CD56^dim^ CD16^+^ and CD56^bright^ CD16^-^ cells in 50 women under IVF treatment and compared between successful IVF and IVF failure with the flowcytometry technique.

**Results:**

Of studied women, 68% did not response to IVF therapy and 32% had successful IVF, the level of CD56^dim^ CD16^+^ cells in women with IVF failure was significantly higher than successful IVF (p<0.0001) but the level of CD56^bright^ CD16^-^ cells was not significantly different between women with IVF failure and successful IVF (p=0.28).

**Conclusion:**

The results of present study demonstrated that the level of NK cells as a risk factor is associated with pregnancy loss in women with IVF failure. However, number of sample in this study is low and further studies with more sample size are needed to be done. We suggest considering treatment option for women undergoing repeated IVF failure with increased percentage of CD56^dim^ cells and the level of peripheral blood NK cell.

## Introduction

Infertility is an increasing medical and social problem. It is estimated that in developed countries 15% of the couples are infertile (1). In vitro fertilization (IVF) which is a high complex technique has become a common and accessible treatment for a wide variety of indications (2). For IVF cycles, the success rate is 40.1% in women less than 35 years to 20.6% in women in the 41-42 years range (3). However, in spite of significant advances, IVF cycle success rates are universally <50% and often <25%. Lost pregnancies are usually due to implantation failure (50-75%) (4). 

Although suggestions have included the failure of at least three cycles of IVF or two cycles of IVF, for repeated IVF failure, there is still no formal consensus definition. Therefore, recently, managing couples with repeated IVF failure and improve IVF outcomes is the urgent need (5). An immunologic basis for unexplained failures has been suspected for many years. The role of the immune system has been confirmed in embryo implantation process (6). Natural killer (NK) cells in both peripheral blood and endometrium are part of the innate immune system. Recently, NK cells have been identified as relevant immunological factors involved in reproductive success. Cellular immune abnormalities with increased peripheral blood NK cell numbers have been shown in women who experienced implantation failures (7).

Of human NK, there are two distinct subsets cells identified by cell surface density of CD56, of these cells approximately 90% are CD56^dim^ and express high levels of CD16 as well as perforin. Approximately 10% of human NK are peripheral blood CD56^bright^ and CD16^-^ NK cells (8). CD56^bright^ and CD56^dim^ cells are functionally and phenotypically different subsets of NK cells. These cells are effective killers and more cytotoxic than other NK subsets. Even resting CD56^dim^ cells are more cytotoxic than CD56^bright^ cells. The increased proportions and cytotoxic activity of blood CD56^dim^CD16^+^ cells and their presence in the endometrium of pregnant women have been related to pregnancy loss (8, 9). 

The role of blood NK cell testing is still unclear, and further research is needed. Therefore, the present study was designed to investigate the relationship between CD56^dim^ cells and CD56^bright^ cells on IVF outcome in infertile women undergoing IVF.

## Materials and methods

This cross sectional study was done from July to December 2013 in Al-Zahra Hospital, Isfahan, Iran. This study was included 50 infertile women who had an indication for IVF treatment. This study was approved by Isfahan University of Medical Sciences. Written informed consent was obtained from all of participants. Advanced maternal age (≥35 years old), history of recurrent miscarriage (due to PCOS, congenital uterine abnormality, thyroid dysfunction, diabetes mellitus or a positive thrombophilia screen), male factor infertility and endometriosis were excluded from the study. Endometrial thickness <8 mm before embryo transfer or fewer than two embryos available for transfer, immune therapy, and taking corticosteroids were the other exclusion criteria. 

Collected information were included; age, body mass index (BMI), parity, previous IVF failures, duration of infertility, and the level of CD56^dim^ cells and CD56^bright^ cells. Blood samples from all the women were taken on days 17-23 of menstrual cycle before the IVF procedure. The flow cytometry technique was done on the fresh blood. Flow cytometry specification is: BD/ Software: Cell Quest (Macintosh-USA)**/** Model: Facscalibw/ Analysis: Four color (FITC, PE, PERCP, APC)/ Laser: Blue 488 nm and Red 635 nm. The peripheral blood cells were washed twice with phosphate-buffered saline before staining, then using color fluorescence -activated cell sorting scan, NK cells were determined after staining with monoclonal antibodies (PARTEC Germany) to CD16 and CD56. To assess IVF outcome pregnancy test was taken from some women had menstruation and had missed period. Successful IVF defined by positive blood b-human chorionic gonadotropin tests performed in the two week after embryo transfer.

**Statistical analysis**

The sample size was calculated to compare two proportions, with two-sided log-rank test, α=0.05, and 80% power. SPSS software (Statistical Package for the Social Sciences, version 20.0, SPSS Inc, Chicago, Illinois, USA) was used to manage and analyze the data. Descriptive data are reported as mean±SD, median [IQR] or n (%) as appropriate. Based on normality test the distribution of CD56^dim^ CD16^+^ cells and CD56^bright^ CD16^-^ cells was not normal in studied subjects. The Student’s *t*-test and Mann-Whitney U-test were used to compare data between women with successful IVF and women with failure IVF. For All probability used tests alpha was set at 5%.

## Results

The mean age of our participants was 32.61±1.70 years. In total, 16 successful IVF cycles (32%) and 34 IVF failure (68%) were observed. The study population characteristics in regard to both successful IVF and IVF failure are shown in table I. Women with successful IVF were significantly younger than women with IVF failure (p=0.003). The mean of BMI in women with successful IVF was less than other women, but this difference was not statistically significant (p=0.36). 

**Figure 1:**
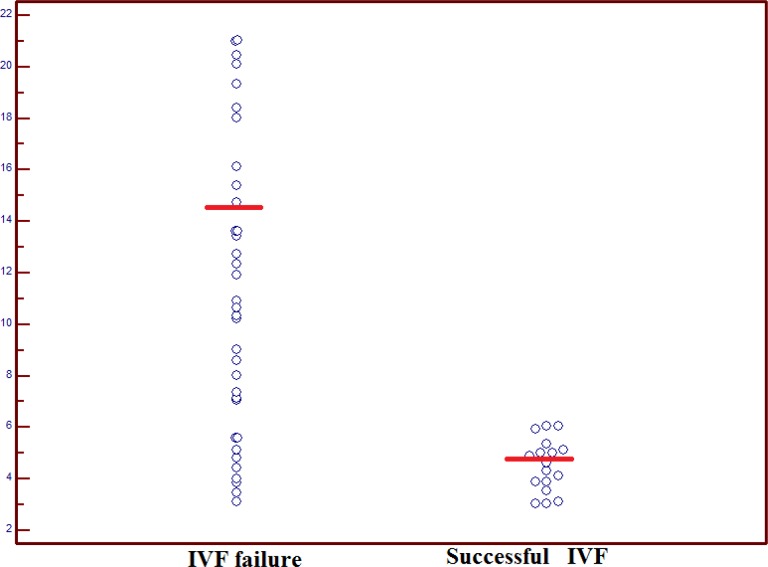
Dot plots of level of CD56^dim ^CD16^+^ cells in women with successful IVF and IVF failure

Previous IVF failures and duration of infertility in women with successful IVF and IVF failure had not statistically significant difference (p=0.12, p=0.61, respectively). The level of CD56^dim^ CD16+ cells in successful IVF group was 4.51±0.98 whereas in IVF failure group it was 13.62±4.63. As it is shown in figure 1, the difference in the level of CD56^dim^ CD16+ cells between the groups was statistically significant (p<0.0001). The level of CD56^bright^ CD16^- ^in successful IVF and IVF failure groups was 0.77±0.22 and 0.71±0.28, respectively.

**Figure 2:**
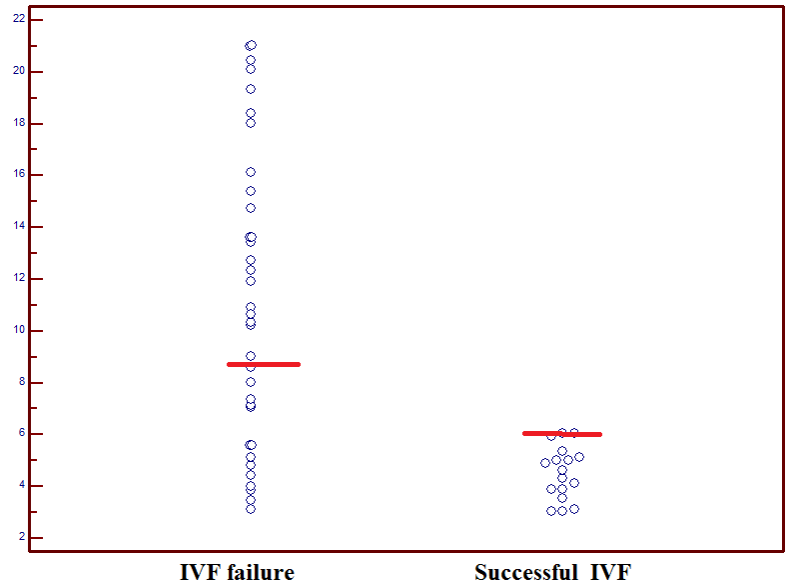
Dot plots of level of CD56^bright^ CD16  cells in women with successful IVF and IVF failure

The difference in the level of CD56^bright^ CD16^-^ cells between groups was not statistically significant (figure 2, p=0.28).

**Table I:** Characteristics and level of CD56^dim^ CD16^+^ and CD56^bright^ CD16^-^ of studied women

	**Successful IVF**	**IVF failure**	**p-value**
Number of patients	16 (32)	34 (68)	-
Age (year)	31.33 ± 2.20	33.11 ± 1.81	0.003*
Body mass index (Kg/m^2^)	22.76 ± 1.32	23.16 ± 1.80	0.36*
Previous IVF failures	1 [0-2]	1.51 [0-3]	0.12†
Duration of infertility (year)	4.41 ± 3.83	4.91 ± 2.72	0.61*
Level of CD56^dim ^CD16^+^cells	4.51 ± 0.98 4.42 [3.70-5.20]	13.62 ± 4.63 13.41 [9.60-18.20]	<0.0001†
Level of CD56^bright^ CD16 cells	0.77 ± 0.22 0.81 [0.53-0.99]	0.71 ± 0.28 0.65 [0.42-1]	0.28†

Data are number (%), mean ± SD, or median [IQR].

* P-value calculated by Student’s *t*-test, and

† Mann-Whitney test.

## Discussion

The results of the present study showed that 68% of treated women did not respond to IVF therapy and 32% had successful IVF. The level of CD56^dim^ CD16^+^ cells in women with IVF failure was significantly higher than successful IVF, but the level of CD56^bright^ CD16^-^ cells was not significantly different between women with IVF failure and successful IVF. Elevated levels of circulating NK cells have been linked to reproductive failure. Significant higher circulating levels of CD56^+^ NK cells than normal fertile controls is demonstrated in women with a history of multiple prior implantation failures after IVF characterized by a negative pregnancy test (10-13). In addition, it is reported that elevated percentages of circulating NK cells during pregnancy have been shown to predict loss of karyotypically normal pregnancies and a normal level associated with loss of embryos that are karyotypically abnormal (14). Aoki *et al* showed that increase in the risk of miscarriage was associated with high peripheral blood NK activity, also Yamada *et al* reported an association between the risk of miscarriage and high peripheral blood NK activity In this study, 50% of the women had increased NK cell levels, which is strikingly similar to the proportion of recurrent miscarriages that are of normal embryonic karyotype (12, 15-16). These data suggested increased numbers and/or activity of circulating blood-type NK related cells can translate into an unfavorable environment at the feto-maternal interface. Another previous study has shown that elevation in the CD3^+^CD56^+^CD16^+^ NKT cell subset was associated with post-implantation pregnancy loss (17). 

As CD3^+^CD56^+^CD16^+^subsets are a major source of pro- inflammatory mediators, they may also have a detrimental effect on implantation (18). Similar to these studies, the results of this study showed that CD56^dim^ CD16^+^ subset is associated with pregnancy loss after IVF. Fukui *et al*, Thum *et al* and Baczkowski *et al* in their studies assessed NK cell in women with infertility who failed to get pregnant (18,19,20). The results of this study reported no difference in percentage of NK cell in women with infertility who failed to get pregnant and those who became pregnant after reproductive technology. In contrast to these results, the results of the current study showed that CD56^dim^ CD16^+^ subset is associated with pregnancy loss after IVF, however, CD56^bright^ CD16^-^ subset was not different between women with infertility who failed to get pregnant and those who became pregnant after IVF. Different results in our study and other studies might be explained by differences in sample size, inclusion criteria or method of analysis. 

Limitations of this study included: 

1. The sample size in the present study was limited and this must be noted in generalizing the findings to the target population. 

2. The number of abortion was not assessed in studied samples in the present study, and in further studies must be noted.

In conclusion, the results demonstrated that the level ofCD56^dim^ CD16^+^ cells was associated with pregnancy loss in women with IVF failure, but the level of CD56^bright^ CD16^-^ cells was not significantly different between women with IVF failure and successful IVF. However, further studies with more sample size are needed to be done. This study suggested considering treatment option for women undergoing repeated IVF failure with increased percentage of CD56^dim^ cells and the level of peripheral blood NK cell.
